# The cause-specific morbidity and mortality, and referral patterns of all neonates admitted to a tertiary referral hospital in the northern provinces of Vietnam over a one year period

**DOI:** 10.1371/journal.pone.0173407

**Published:** 2017-03-10

**Authors:** Merinda Miles, Khu Thi Khanh Dung, Le Thi Ha, Nguyen Thanh Liem, Khu Ha, Rod W. Hunt, Kim Mulholland, Chris Morgan, Fiona M. Russell

**Affiliations:** 1 Faculty of Medicine, Nursing and Health Sciences, Monash University, Melbourne, Australia; 2 National Hospital of Pediatrics, Hanoi, Vietnam; 3 Murdoch Childrens Research Institute, The Royal Children’s Hospital, Melbourne, Australia; 4 Department of Neonatal Medicine, The Royal Children’s Hospital, Melbourne, Australia; 5 Centre for International Child Health, Department of Paediatrics, The University of Melbourne, Melbourne, Australia; 6 Menzies School of Health Research, Darwin, Australia; 7 London School of Hygiene and Tropical Medicine, London, United Kingdom; 8 Centre for International Health, Burnet Institute, Melbourne, Australia; 9 Melbourne School of Population and Global Health, The University of Melbourne, Melbourne, Australia; 10 School of Public Health and Preventive Medicine, Monash University, Melbourne, Australia; Centre Hospitalier Universitaire Vaudois, FRANCE

## Abstract

**Objective:**

To describe the cause-specific morbidity and mortality, and referral patterns of all neonates admitted to a tertiary referral hospital in the northern provinces of Vietnam.

**Design:**

A prospective hospital based observational study.

**Setting:**

The Neonatal Department, National Hospital of Pediatrics, Hanoi, Vietnam.

**Patients:**

All admissions to the Neonatal Department over a 12 month period.

**Main outcome measures:**

Cause-specific morbidity and mortality; deaths.

**Results:**

There were 5064 admissions with the commonest discharge diagnoses being infection (32%) and prematurity (29%). The case fatality ratio (CFR) was 13.9% (n = 703). Infection (38%), cardio/respiratory disorders (27%), congenital abnormalities (20%) and neurological conditions (10%) were the main causes of death. Of all the deaths, 38% had an admission weight ≥2500g. Higher CFR were associated with lower admission weights. Very few deaths (3%) occurred in the first 24 hours of life. Most referrals and deaths came from Hanoi and neighbouring provincial hospitals, with few from the most distant provinces. Two distant referral provinces had the highest CFR.

**Conclusions:**

The CFR was high and few deaths occurred in neonates <24 hours old. The high rates of infection call for an improvement in infection control practices and peripartum antibiotic use at provincial and tertiary level. Understanding provincial hospital capacity and referral pathways is crucial to improving the outcomes at tertiary centres. A quality of care audit tool would enable more targeted interventions and monitoring of health outcomes.

## Introduction

The slow decline in neonatal mortality impeded the attainment of Millennium Development Goal 4 for many countries. Globally, most neonatal deaths are due to prematurity (36%) infections (23%), asphyxia (23%), and congenital malformations (10%) [1, 2]. Data on cause-specific mortality is important for health planning as specific interventions are available, even in the most poorly resourced settings, to prevent and treat many of these conditions [3].

The postnatal period has been identified as a significant gap in service provision [4]. The recognition and management of common neonatal conditions requires clinical expertise and training, access to suitable equipment, and efficient referral pathways [5, 6]. To ensure health care is of sufficient quality, it is important to monitor outcomes of care provided [7], and then use this information to strategically determine which interventions should be prioritized.

Vietnam is a lower-middle income country (LMIC) and has shown substantial reductions in child mortality. The Neonatal Mortality Rate (NMR) is 11 per 1,000 live births in Vietnam, although population based studies show considerable regional variation [8, 9]. In Vietnam, over 90% of births occur in health facilities [10]. However most published data on neonatal health in Vietnam is community based [11–14]. Community based studies from rural northern Vietnam have linked neonatal mortality with ethnicity, place of birth and a poor referral system[15]. Provincial hospitals with newborn care units are often not equipped to treat or have training to care for many neonatal conditions (Khu, pers. comm.). Furthermore, families frequently bypass their local hospital and self-refer to major hospitals that are far away; this can result in a deterioration in the condition of neonates as safe transport systems are underdeveloped (Khu, pers. comm.). There are few published data on cause-specific neonatal morbidity and mortality, despite most deaths occurring in the hospital setting, particularly in northern Vietnam. The aim of this study is to describe the cause-specific morbidity and mortality, and referral patterns of all admissions to the largest neonatal unit in northern Vietnam.

## Methods

### Study site

The National Hospital of Pediatrics (NHP), Hanoi, is the leading pediatric department in Vietnam [16]. It is a tertiary referral centre for northern Vietnam and all neonates admitted to the Neonatal Department are born off-site. There are 25 provinces in northern Vietnam with a total population of approximately 37, 285,400 [17]. Each province has provincial and district hospitals. For convenience, hospital referring from Hanoi Province in this study are categorised as provincial hospitals. The Neonatal Department has 140 beds but can have a bed occupancy of up to 270 patients. The department has a nurse to patient ratio of 1:11. All doctors undergo neonatal training.

The Neonatal Department has access to advanced diagnostics including molecular microbiology, genetic testing and radiological imaging. Treatment options include: oxygen, artificial ventilation, intravenous and nasogastric therapy, exogenous surfactant, antibiotics, phototherapy, exchange transfusion, parenteral nutrition, and surgical procedures for congenital anomalies and cardiac defects.

### Study design

This was a 12-month prospective hospital based observational study of all neonates admitted to the Neonatal Department, NHP, between the 1^st^ of July, 2011 and 30^th^ of June, 2012. Outcomes were monitored until the 17^th^ of August, 2012. In this study all participants are termed “neonates”, for convenience, even though some may have been older than 28 days.

### Data collection

Demographic and clinical details were extracted from each neonate’s medical record. Admission weight was collected as reliable birth weight information was not available for all neonates as all were outborn and birthweight was either not recorded or not always available. Gestational age is not routinely recorded.

Discharge diagnoses were categorised using the Perinatal Society of Australia and New Zealand Mortality classification system (PSANZ-NDC), 2009 [18] by co-author (MM) using the primary discharge diagnosis. These classifications included congenital abnormality, extreme prematurity, prematurity, cardio/respiratory disorders, infections, neurological, gastrointestinal, other, and unknown.Neonates who had a discharge diagnosis of “prematurity” were classified as “extreme prematurity” if the admission weight was ≤600g as per PSANZ-NDC, or “premature” if no other discharge diagnosis was recorded. Neonates who had a discharge diagnosis of “jaundice” were classified as “other” if no other diagnostic information was available.

An outcome of death was recorded for any neonate who died, or was discharged home to die. All deaths were identified by a Vietnamese neonatal doctor (LTH), who attended the ward’s handover each week-day and reviewed all medical records. Deaths were classified using the PSANZ-NDC system. Pneumonia was classified as “”Infection: Other” unless the aetiology (acquired or congenital) was known. For “extreme prematurity” classifications, the admission weight was checked to ensure it was <600g. In cases where the admission weight was >600g, cause of death was reclassified as “unknown” if no other discharge diagnosis was recorded.

### Data management

Data for all admissions and deaths were entered in Excel and EpiData version 3.1, respectively, then transferred to STATA Version 12.1 for cleaning and analysis. Any discrepancies were re-checked against the medical record.

### Statistical analysis

Continuous data were summarised as medians and inter-quartile ranges. Categorical data were summarised by number and proportion of cases for each variable. Categorical data were compared using a Chi Square Test, or Fisher’s exact test, where appropriate. STATA Version 12.1 was used to map home province.

### Ethical approval

Ethics approvals were gained from the NHP Ethics Committee, Hanoi, and the Monash University Health Research Ethics Committee, Melbourne, Australia. No individual consent was obtained as this study was deemed by the local ethics committee as an audit, with individual consent not being required.

## Results

There were 5064 neonates admitted to NHP, and 703 died (CFR 13.9%). Table 1 shows the features of all admissions and deaths. More than half of all admissions (56.7%) were referred from provincial hospitals and 24% were self-referred. Most deaths (87.1%) were referred from provincial hospitals, with CFR of 21.3%, (n = 612). The CFR for admissions that had been self-referred (n = 1214) was lower (3.4%). Nearly half of admissions (49.9%) and 88.5% of neonates who subsequently died, were transferred by ambulance.

**Table 1:** Demographic, referral, and clinical features of all admissions and deaths to the Neonatal Department, National Hospital of Pediatrics, between 1^st^ July 2011 and 30^th^ June 2012.

Demographic features		Admissions (n = 5064)	Deaths (n = 703)	CFR
		n (%)^1^	n (%)^1^	
**Gender**	Male	3388 (66.9%)	446 (63.4%)	13.2%
	Female	1673 (33.0%)	256 (36.4%)	15.3%
	Unstated	3 (0.1%)	1 (0.1%)	33.3%
**Referral centre**	Provincial hospital	2871 (56.7%)	612 (87.1%)	21.3%
	District hospital	110 (2.2%)	36 (5.1%)	32.7%
	Self-referral	1214 (24%)	41 (5.8%)	3.4%
	Other	2 (0.03%)	1 (0.1%)	50%
	Unknown	867 (17.1%)	13 (1.8%)	1.5%
**Mode of transport**	Ambulance	2529 (49.9%)	622 (88.5%)	24.6%
	Car	849 (16.8%)	21 (3%)	2.5%
	Motorbike	1104 (21.8%)	3 (0.4%)	0.3%
	Unknown	582 (11.5%)	57 (8.1%)	9.8%
**Median admission weight (IQR)**^2^		2600g (1850-3200g)	2100g (1470–2870)g	
**Admission weight**	<1000g	63 (1.2%)	32 (4.6%)	50.8%
	1000-1499g	490 (9.7%)	146 (20.8%)	29.8%
	1500-2499g	1685 (33.3%)	256 (36.4%)	15.2%
	≥2500g	2721 (53.7%)	267 (38%)	9.8%
	Unknown	105 (2.1%)	2 (0.28%)	1.9%

^1.^Unless stated otherwise

^2.^Median and interquartile range

For those neonates who died, the median survival time from admission was 5 days (IQR 1–14 days). Most deaths (81.7%) occurred within the first 28 days of life. Only 3% (n = 21) of all deaths in this facility occurred within the first 24 hours of life, while 45.2% of all deaths were late neonatal deaths (between 7 and 28 days). Over one-third (38%) of neonates who died had an admission weight of ≥2500g. Although there were fewer deaths in number, the CFRs were highest for neonates <1000g (33.7%).

**Fig 1:**
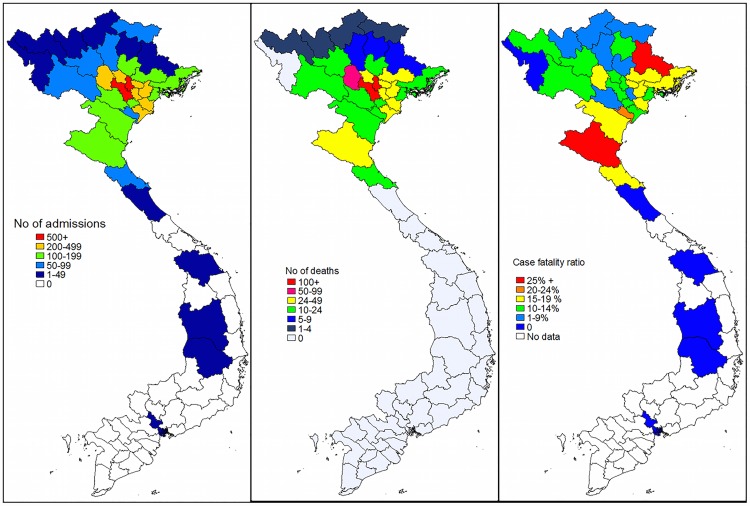
The number of admissions (a), deaths (b), and case fatality ratio (c), to the Neonatal Department, National Hospital of Pediatrics, Hanoi, 1st July 2011 to 30th June 2012, by home province.

Most referrals came from Hanoi Province and its neighbouring provinces (Fig 1). Almost half of the self-referrals came from Hanoi Province (n = 548, 45%). There were few admissions and deaths from provinces that were more distant. However the CFRs were high from two distant provinces: Nghe An Province (CFR = 26.8%) and Lang Son Province (CFR = 25%).

### Cause-specific morbidity

**Fig 2:**
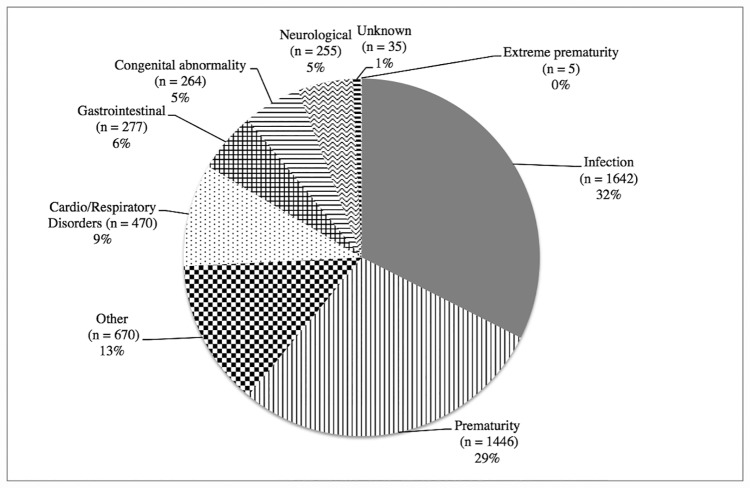
Discharge diagnoses^1^ of all admissions to the Neonatal Department, National Hospital of Pediatrics, Hanoi, 1st July 2011 to 30th June 2012 (n = 5064).

The two commonest discharge diagnoses were infection (32%) and prematurity (29%) (Fig 2). “Other causes” were the third commonest category (13%). The majority of these “other causes” had signs of jaundice (9% of all admissions) but no other diagnostic information was performed. Of all the cases with jaundice (n = 458), 12.9% had the diagnosis of kernicterus. Congenital abnormalities accounted for 5% of all admissions.

### Cause-specific mortality

**Fig 3:**
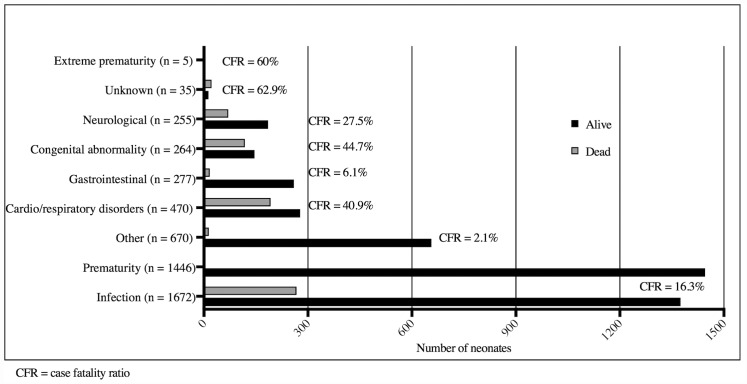
Discharge diagnoses and cause-specific case fatality ratios by PSANZ-NDC diagnostic category, Neonatal Department, National Hospital of Pediatrics, Hanoi, 1^st^ July 2011 and 30^th^ June 2012 (n = 5064).

**Fig 4:**
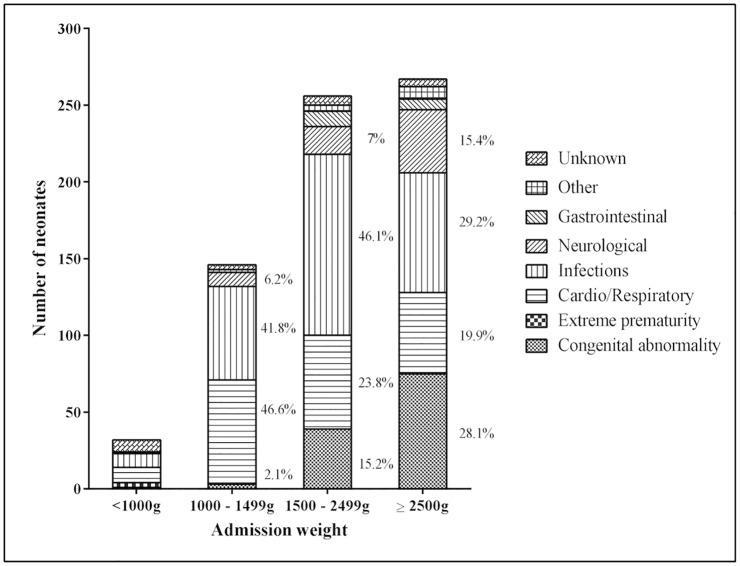
Cause-specific mortality by admission weight, Neonatal Department, National Hospital of Pediatrics, Hanoi, between 1^st^ July 2011 and 30^th^ June 2012.

The commonest primary causes of death were infection (38%), cardio/respiratory disorders (27%), congenital abnormalities (17%) and neurological disorders (10%) (Fig 3). Excluding unknown causes of death and extreme prematurity (CFR 60%), congenital abnormalities had the highest CFR (44.7%), followed by cardio/respiratory disorders (40.9%). For neonates who died and had admission weights ≥ 1500g, the next most common causes of death after infection, were cardio/respiratory disorders and congenital abnormalities (Fig 4).

Infection was the commonest cause of death irrespective of admission weight (Figs 3 and 4), and had a CFR of 16.3% (Fig 3). Infections were a contributory factor for an additional 35 (5%) deaths. Of the 267 infections that were fatal, 21.7% were in neonates admitted within the first 24 hours of life and a further 36.3% were admitted between days 1–6 of life. Of the 302 fatal infections (both primary and contributory), a pathogen specific aetiology was identified for 122 (40.4%) infections. *Klebsiella* spp. was the commonest cause (n = 26, 21%), while fungal infections (including *Candida* spp) accounted for 10% (n = 12) of these infections.

Congenital infections were responsible for 16% of infections. Neonatal tetanus occurred in one fatal case. This ethnic minority infant was born at home, and the umbilical cord was cut with scissors. No further information regarding antenatal or postnatal care was recorded. Thirty-eight deaths were classified as congenital rubella syndrome, comprising 31% of the infections with a known aetiology.

Hyaline-Membrane Disease/Respiratory Distress Syndrome (HMD/RDS) caused 15.5% of all deaths. Almost half (49.9%) were in neonates admitted to the NHP within 24 hours of life. Over one quarter (28.4%) of neonates who died from HMD/RDS died within 24 hours of admission. S1 Fig shows 84.9% of deaths from HMD/RDS were in neonates with admission weights 1000-2400g. Pneumothoraces caused 7.5% of all deaths. Almost all of these neonates (96.2%) had been referred from provincial hospitals. One quarter of these neonates died within the first 24 hours of admission.

Cardiovascular abnormalities were the cause of 78% of congenital abnormality deaths, representing 13.1% of deaths. Hypoxic ischaemic encephalopathy (HIE) (n = 39) accounted for 6.4% of deaths, and intracranial haemorrhages (n = 23) accounted for 3.3% of deaths. Almost all (94.9%) deaths from HIE were in neonates with an admission weight ≥1500g and 23.1% died within 24 hours of admission.

## Discussion

Neonates admitted to tertiary health facilities represent an important subgroup that have a high risk of mortality. We identified areas where attention could be focused to improve outcomes in the largest neonatal department in northern Vietnam. We found that infection and prematurity were the commonest reasons for admission. In contrast, a study from a tertiary referral hospital in Ho Chi Minh City, in southern Vietnam, found that prematurity, asphyxia, and congenital malformations were underrepresented in admissions, and found one quarter of the diagnoses did not require specialist care [19], which may indicate differences in referral patterns to NHP. The commonest causes of death in our study were infection, cardio/respiratory disorders, and congenital abnormalities, accounting for 82% of deaths. This is similar to the tertiary referral hospital for Southern Vietnam which showed major causes of death were congenital abnormalities, severe infection and prematurity, comprising 80% of all deaths [20].

The CFR for newborn care units in LMICs is highly variable [21–25] and may relate to socio-cultural or health system factors affecting timeliness of referral, disease burden, and quality of care provided.

As NHP is a tertiary referral hospital, very sick neonates are referred for specialised care, so it is unsurprising that we found a CFR of 13.9%. The difference with the tertiary referral centre in Southern Vietnam, where the CFR was 5%, demonstrates significant variation even within countries [20].

Our study has some important differences from population-based studies of neonatal mortality, consistent with this hospital’s role as a tertiary centre rather than a place of birth. For example, intra-partum related deaths represented a low percentage of deaths, given this aetiology has been identified as one of the commonest causes of neonatal death worldwide [1]. We also found very few deaths (3%) occurred in the first 24 hours of life at NHP. Both community data from Northern Vietnam which showed 58.6% of deaths occurred within the first 24 hours [15], and that global data suggest the risk of death is greatest on the first day of life, suggests that for northern Vietnam, the majority of these neonatal deaths occur outside the tertiary system [1]. While neonatal deaths due to infections and preterm complications have effective interventions for LMICs, solutions for intra-partum complications are more challenging and require strengthening of all levels of the health system [1, 26, 27].

As all admissions to NHP are born elsewhere, the decision to present to NHP for admission is influenced by many factors. However, one critical factor is the rapid identification and referral of complications in the first days after birth. The referral pattern in this study suggests that the sickest neonates are cared for initially at local provincial hospitals, which is consistent with the referral protocol of Vietnam. However, there were also many self-referrals (that is, by the family themselves) and these had a low CFR. Another study in Vietnam suggests that bypassing district hospitals for health care is common [28]. If many of the self-referrals presenting to NHP have minor conditions, they may be contributing to an unnecessary admission overload, especially if they can be cared for by a nearby hospital. The extremely high bed occupancy rate at NHP, exacerbated by insufficient human resources, may impact on the quality of care provided. A study in India found that admission overload had a negative impact on CFR [21]. Understanding referral pathways and admission criteria is vital to reduce patient overload at the tertiary level.

Our findings highlight the need to further understand the care provided at provincial hospitals and the adequacy of the referral system. We found few admissions and deaths from provinces that were more distant from NHP, and disparate CFRs by referral province. In addition, we found neonates presenting to NHP with the preventable and treatable condition of kernicterus. HMD/RDS was the second commonest cause of mortality. It is not known whether exogenous surfactant was administered prior to referral, or whether pre-delivery corticosteroids were administered. Both findings raise concerns regarding the adequacy of pre-referral treatment at district or provincial levels. Previous studies have shown that prevention and early management of prematurity (including HMD/RDS) at the site of birth is important to survival [29].

Several of our findings have potential implications for the quality of care at NHP. Our data suggest hospital acquired infections are common, given the organisms involved, and the timing of the onset of infection (data not shown). This is consistent with data from a central Vietnamese neonatal unit, which showed that 80% of infections were nosocomial [30]. To reduce hospital acquired infection rates, enhanced infection control practices are being implemented within NHP. In addition, these guidelines should be implemented in all hospitals. We found pneumothoraces to be relatively common. Further understanding on the timing of the event and the type of supplemental ventilation used is required to ascertain whether less invasive forms of ventilation may be warranted. This again highlights the need for understanding and improving the quality of care at referral hospitals.

In this study, 38% of deaths occurred in neonates with admission weight of ≥2500g. This is similar to other LMICs [23]. This pattern may be partially explained by a larger caseload of congenital abnormalities of normal birth weight being transferred to NHP, and selective referral practices for very low and extremely low birth weight neonates. We found there were few very low birth weight neonates referred, but those that were referred had a high CFR. Smaller, more premature neonates may not be referred due to their likely poor prognosis. Worldwide data consistently shows that the lower the birth weight, the higher the risk of a poor outcome [31, 32]. The introduction of basic standards of neonatal care in the highlands of Papua New Guinea reduced CFR in very low birth weight neonates by 56% [33].

In our study, 17% of deaths were due to congenital abnormalities and many of these were due to cardiac defects. This is consistent with NHP’s role as a tertiary referral centre in providing expert diagnostic and treatment facilities. A large rubella outbreak resulted in many cases of congenital rubella syndrome [34]. Rubella vaccination has recently been implemented in Vietnam and should, over time, reduce the burden of congenital rubella syndrome.

Although prematurity is not in itself a disease, it is a pathological state, and in this study we were unable to fully assess the role of prematurity to neonatal morbidity and mortality, despite prematurity being the commonest reason for admission. For future studies, it would be beneficial to provide an assessment of gestational age, although the additional workload needs to be considered.

There is increasing evidence in LMICs that auditing improves the quality of neonatal care. A systematic review of perinatal audits in LMICs demonstrated a 30% (95% CI, 21%-38%) reduction in perinatal deaths post audit introduction [35]. Our study demonstrates both the feasibility and the difficulties of implementing a mortality and morbidity database in a busy neonatal unit using existing clinical staff. In addition, the lack of testing facilities to diagnosis the common clinical sign of jaundice is a limitation. In this study we only recorded the primary cause of mortality but would recommend that secondary causes also be documented. Further training would also be required to avoid misclassification of some conditions and completeness of data collection. In this study we classified neonates with evidence of congenital rubella syndrome as “infection” as many did not have obvious abnormalities but had evidence of inflammation. This data could be further improved and more complete if collected by a trained audit committee, which should determine diagnostic classifications and also prioritize interventions [11].

## Conclusions

We have provided a baseline assessment of the outcomes of neonates admitted to the largest tertiary referral hospital in northern Vietnam and have identified a number of areas for intervention so that further improvement in neonatal outcomes can be obtained. This study highlights the need for greater research into pre-referral care at first- and second-line facilities and for investigation of quality improvement activities such as audits to benefit neonatal care in all health facilities.

## Supporting information

S1 Fig Specific cardio/respiratory causes of mortality by admission weight, Neonatal Department, National Hospital of Pediatrics, Hanoi, 1^st^ July 2011 to 30^th^ June 2012. (TIF)

S1 Dataset Data collected from Neonatal Department, National Hospital of Pediatrics, Hanoi, 1^st^ July 2011 to 30^th^ June 2012. (XLSX)
