# Supplementary figures and images for: The cause-specific morbidity and mortality, and referral patterns of all neonates admitted to a tertiary referral hospital in the northern provinces of Vietnam over a one year period
Source: PLoS One. 2017 Mar 10;12(3):e0173407. doi: 10.1371/journal.pone.0173407 (PMC5345801; doi:10.1371/journal.pone.0173407)

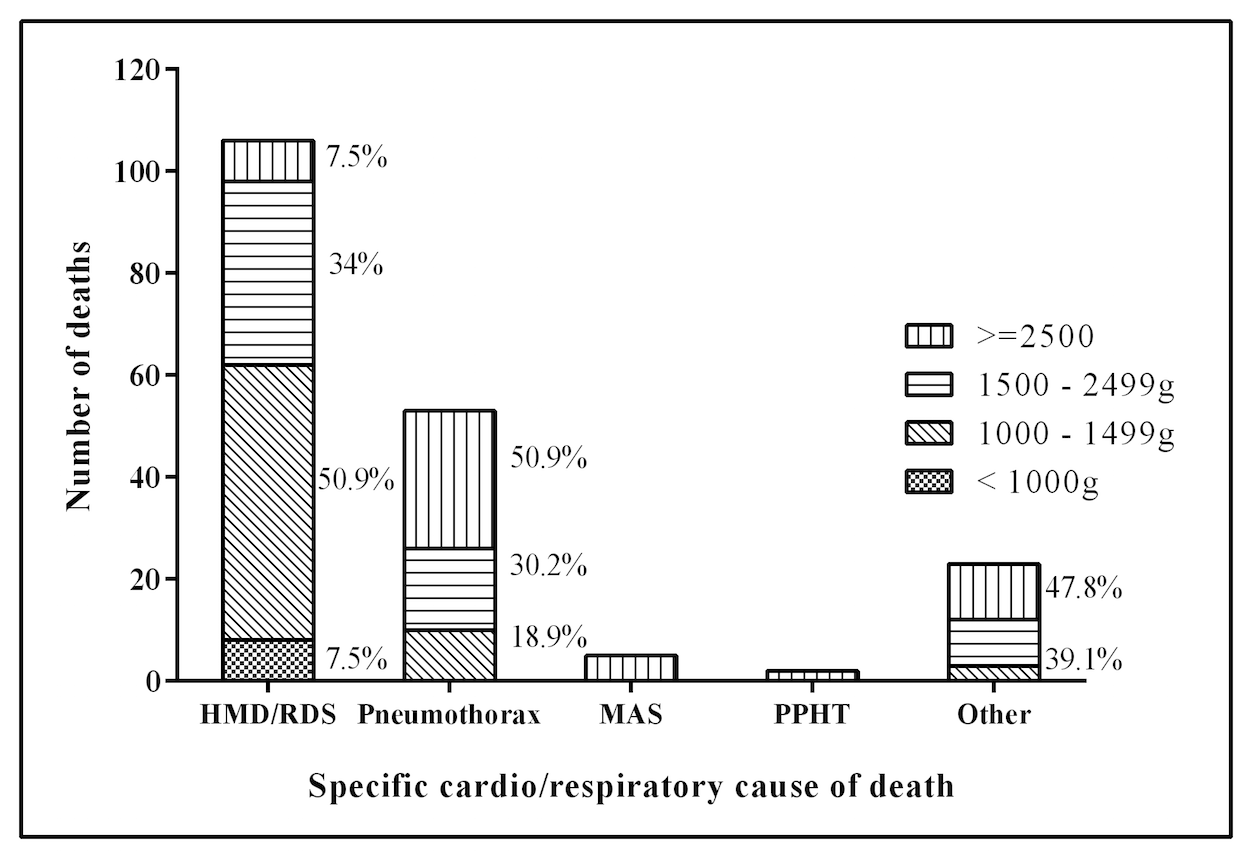

Supplement: S1 Fig — (TIF) [file pone.0173407.s001.tif]
